# The phosphodiesterase 5 inhibitor tadalafil has renoprotective effects in a rat model of chronic kidney disease

**DOI:** 10.14814/phy2.14556

**Published:** 2020-09-05

**Authors:** Natsumi Tomita, Yuji Hotta, Aya Naiki‐Ito, Kana Hirano, Tomoya Kataoka, Yasuhiro Maeda, Satoru Takahashi, Kazunori Kimura

**Affiliations:** ^1^ Department of Hospital Pharmacy Graduate School of Pharmaceutical Sciences Nagoya City University Nagoya Japan; ^2^ Department of Experimental Pathology and Tumor Biology Graduate School of Medical Sciences Nagoya City University Nagoya Japan; ^3^ Department of Clinical Pharmaceutics Graduate School of Medical Sciences Nagoya City University Nagoya Japan; ^4^ Center for Joint Research Facilities Support Fijita Health University Toyoake Japan

**Keywords:** chronic kidney disease, phosphodiesterase 5 inhibitors, renoprotection, tadalafil

## Abstract

Phosphodiesterase 5 inhibitors are widely used to treat erectile dysfunction and lower urinary tract symptoms with benign prostatic hyperplasia. Recent studies have indicated the renoprotective effects of this class of compounds. Whether renoprotection depends on blood pressure reduction remains controversial. In this study, we investigated the renoprotective effects of the phosphodiesterase 5 inhibitor, tadalafil, in a rat model of high‐salt induced kidney injury with hypertension. Dahl salt‐sensitive rats were fed a normal diet, high‐salt (8% sodium chloride) diet, or high‐salt diet with oral administration of either low‐ or high‐dose tadalafil (1 and 10 mg kg^−1^ day^−1^, respectively). Serum creatinine, urinary protein, and blood pressure were measured at baseline and after 8 weeks, at which point the rats were examined for glomerular injury and fibrosis. PAI1 mRNA levels were also evaluated. After 8 weeks, blood pressure, serum creatinine, and urinary protein levels were significantly higher in the high‐salt group than those in the normal‐salt group. Serum creatinine and urinary protein were significantly lower in both tadalafil groups than those in the high‐salt group, while only high‐dose tadalafil affected blood pressure. In addition, glomerulosclerosis and α‐smooth muscle actin expression significantly decreased in both tadalafil treatment groups. PAI1 mRNA increased significantly in the high‐salt group but decreased in both tadalafil‐treated groups. Our results indicated that both low‐ and high‐dose tadalafil prevented fibrosis and glomerular injury in a chronic kidney disease rat model. Mechanistically, these effects may be associated with PAI1 expression and glomerular structure protection.

## Graphical abstract

A PDE5 inhibitor, Tadalafil is renoprotective by preventing glomerular injury and fibrosis from high blood pressure. This study suggest that Inhibition of PDE5 might be effective to delay the CKD progression.

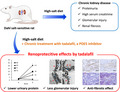

## 1 INTRODUCTION

Chronic kidney disease (CKD) is a major global health concern and often associated with other conditions, thus, increasing comorbidities (Goleg, Kong, & Sahathevan, 2014; Japanese society of nephrology (JSN), 2018; Szczech & Lazar, 2004). In particular, patients with diabetes, hypertension, and atherosclerosis have a high risk of progressing to end‐stage kidney disease (ESKD) (Hanafusa, Nakai, Iseki, & Tsubakihara, 2015; KDIGO, 2012; KDIGO, 2012). Since ESKD remains as an urgent health concern, novel therapeutic targets to alleviate and/or delay the progression of CKD are warranted.

Hypertension is a risk factor for CKD progression. It accelerates the development of ESKD and is closely correlated with renal dysfunction. Many patients with CKD and hypertension are salt‐sensitive (KDIGO; Tozawa et al., 2003) and restricting their daily salt intake is an effective strategy to prevent blood pressure (BP) elevation. Common antihypertensive drugs such as angiotensin‐converting‐enzyme inhibitors, angiotensin Ⅱ receptor blockers, and calcium channel blockers are known to be renoprotective (Hollenberg, 2001; KDIGO). However, these treatments have little effect on reducing ESKD. Moreover, the calcium channel blocker, amlodipine, prevents BP elevation but not proteinuria and kidney injury in a salt‐sensitive model of hypertension (Takai, Jin, Sakonjo, & Miyazaki, 2010).

Phosphodiesterase 5 (PDE5) inhibitors are used to treat erectile dysfunction and lower urinary tract symptoms with benign prostatic hyperplasia and are effective against renal dysfunction (Fang et al., 2013; Li et al., 2012; Rodríguez‐Iturbe et al., 2005; Stegbauer et al., 2013). Daily treatment with PDE5 inhibitors could attenuate kidney injury and BP elevation in models of diabetic nephropathy, renal ischemia‐reperfusion injury, and CKD (Fang et al., 2013; Li et al., 2012; Rodríguez‐Iturbe et al., 2005; Stegbauer et al., 2013). Further, the inhibition of nitric oxide (NO)/cyclic guanosine monophosphate (cGMP) signaling in the kidney can cause renal dysfunction (Fang et al., 2013; Schmidt & Baylis, 2000), and PDE5 inhibitors prevent cGMP degradation, thus, increasing its concentration.

PDE5 inhibitors promote vascular smooth muscle relaxation, and consequently, bring about a pronounced lowering of BP. Therefore, they may be novel effective therapies for renal dysfunction, since they increase cGMP levels (Fang et al., 2013; Rodríguez‐Iturbe et al., 2005; Stegbauer et al., 2013). However, it remains uncertain whether they are useful for treating renal dysfunction with salt‐sensitive hypertension. In this study, we investigated whether tadalafil, a PDE5 inhibitor, was effective in treating a rat model of salt‐sensitive hypertension and kidney injury induced by excessive salt intake.

## 2 MATERIALS AND METHODS

### 2.1 Experimental protocols

Eight‐week‐old male Dahl salt‐sensitive rats (DIS/EiS, Japan SLC Inc.) were housed in a room with controlled temperature, humidity, and a 12 hr light/dark cycle with free access to normal water. We divided the rats into the following four groups (*n* = 5–7), which were treated as indicated: normal salt (NS; 0.3% sodium chloride [NaCl]‐containing rodent diet CE‐2 (CLEA Japan, Inc.), high salt (HS; 8% NaCl + CE‐2), and high salt plus low‐ (TL) or high‐dose tadalafil (TH; 1 and 10 mg kg^−1^ day^−1^, respectively, Nippon Shinyaku Co., Ltd.). An NS + TH (10 mg kg^−1^ day^−1^) group was treated using the same protocols (Table S1).

The TL and TH groups were treated orally with tadalafil in 0.5% of hydroxypropyl methylcellulose once daily for 8 weeks, while the NS and HS groups were treated with 0.5% of hydroxypropyl methylcellulose. BP was measured using the tail‐cuff method (BP‐98A‐L, Softron Co., Ltd.) at 0 and 8 weeks. Briefly, rats were warmed at 37°C in an animal holder and kept for approximately 30 min to calm. BP was measured three times and the mean was calculated. Metabolic cages were used for 24 hr urine collection at 0 and 8 weeks. In addition, blood samples were acquired from the tail vein at week 0 and from the inferior vena cava under 2% of isoflurane anesthesia at week 8. The kidneys were harvested following euthanasia. The two halves of the right kidney were used for histopathological assessment and electron microscopy. The cortex and medulla of the left kidney were separated and analyzed using real‐time polymerase chain reaction (PCR). All animal procedures were approved by the Ethics Committee of Nagoya City University and performed according to the guidelines of the National Institutes of Health Science of Japan (H29‐P‐05).

### 2.2 Blood and urine analysis

Blood and urine were collected at 0 and 8 weeks. Serum creatinine (SCr), blood urea nitrogen (BUN), and the urinary protein to Cr ratio (UPC) were determined to evaluate the kidney function using enzymatic methods for SCr and BUN, and enzymatic and colorimetric methods for UPC. Serum Na, Cl, and K levels and total urinary Na, Cl, and K excretion over 24 hr were determined using the ion‐sensitive electrodes method. All measurements were conducted by Fujifilm Monolith Co., Ltd.

### 2.3 Real‐time PCR analysis

Briefly, total RNA was extracted from the kidney cortex and medulla using an RNeasy Mini Kit (Qiagen). RNA concentration and quality were measured using spectrophotometric analysis at 260 and 280 nm. A Rever Tra Ace kit (TOYOBO Co., Ltd.) was used to reverse transcribe 1 µg total RNA to cDNA. Real‐time PCR was performed using a CFX Connect Real‐Time PCR Detection System (Bio‐Rad Laboratories, Inc.) with KAPA SYBR FAST qPCR Master Mix (2×; Nippon Genetics Co., Ltd.). Relative expression levels were calculated using the ΔΔCt method with β actin as a reference. Primer sequences are listed in Table 1. The specificity of each primer was verified using a dissociation curve.

**Table 1:** Primer sequences for real‐time PCR

mRNA	Sequence
PAI1	
Sense	5′‐GGAGAGGCACACCAAAGGTAT‐3′
Antisense	5′‐GTGCTGGCCTCTAAGAAGGG‐3′
β actin	
Sense	5′‐TGTGTGGATGGTGGCTCTATC‐3′
Antisense	5′‐CATCGTACTCCTGCTTGCTGATC‐3′

### 2.4 Histopathological analysis

Kidney tissue was fixed in 10% of formalin for 2 days and placed in 70% of ethanol. The tissues were embedded in paraffin, and then, serially sectioned to 3‐μm thick samples. After deparaffinization and rehydration, the kidney sections were examined using periodic acid Schiff (PAS) and Azan staining.

To evaluate the level of glomerulosclerosis, 30 glomeruli in each kidney were analyzed in PAS‐stained sections and graded as follows based on the severity of glomerular injury: grade 0, no sclerosis; grade 1, <25%; grade 2, 25% to 50%; grade 3, 50% to 75%; grade 4, >75%. The glomerulosclerosis index (GSI) was calculated using the following formula, which is widely used (Forbes et al., 2003; Wu et al., 1997);

$$\text{GSI}=\left[\left(0\times N0\right)+\left(1\times N1\right)+\left(2\times N2\right)+\left(3\times N3\right)+\left(4\times N4\right)\right]/30$$

where Nx is the number of glomeruli scored as x.

Paraffin sections were also used for immunohistochemical staining using an antibody against α smooth muscle actin (αSMA; 1:200; Dako), as reported previously (Sagawa et al., 2015). As αSMA is a myofibroblast marker, it indicates the presence of active fibroblasts, which are known to induce extracellular matrix production. Five areas in the outer medulla were randomly selected (excluding the vasculature) and examined at 20× magnification using a BZ‐9000 Fluorescence Microscope (Keyence). The αSMA‐positive area of each kidney section was calculated using the following formula: positive area (%) = (αSMA‐positive area)/(total image area). The mean positive area from five images per sample was used for analysis. Serial sections were also observed using Azan staining to confirm extracellular matrix deposition.

### 2.5 Electron microscopy

The kidney cortex was fixed with a mixture of 2.5% glutaraldehyde and 2% paraformaldehyde (Nisshin EM) in 0.1 M of phosphate buffer. Then, the tissue was washed, post‐fixed with 2% of osmium tetroxide (OsO_4_; Nisshin EM), dehydrated, embedded in Quetol 651 (Nisshin EM), and sectioned to 80–100 nm thickness. Tissue samples were subsequently stained with uranyl acetate and observed using a JEM‐1400Plus Transmission Electron Microscope (JEOL Ltd.).

For scanning electron microscopy, samples were fixed with 2% of OsO_4_, dehydrated, and then, freeze‐fractured in various alcohols. After supercritical drying, the samples were coated with Os and observed using a S‐4800 Field Emission Scanning Electron Microscope (Hitachi).

### 2.6 Statistical analysis

Data are expressed as the mean ± *SEM*. Comparisons were made using one‐way analysis of variance and Tukey's test. Data with a *p* < .05 were considered statistically significant.

## 3 RESULTS

### 3.1 General characteristics

There were no significant differences in body weight between the groups before or after the experiment (Table 2a). Serum electrolyte levels remain unchanged (Table 2b), while urinary Na and Cl excretion over 24 hr were significantly larger in the HS, TL, and TH groups than in the NS group (Table 2c). There were no statistical differences in the 24 hr urine volume between the different groups (Table 2d). BP increased in the HS, TL, and TH groups and was significantly higher at 8 weeks than that in the NS group (*p* < .01, Table 3). BP levels in the HS and TL groups were comparable, while those in the TH group were significantly lower than the HS group. The heart rate remained unchanged among all groups (Table 3). In addition, the BP levels and heart rates of rats fed a normal diet and treated with high‐dose tadalafil were not significantly different from those in the NS group (Table S1a).

**Table 2:** Body weights and serum and urine electrolyte levels at baseline and after 8 weeks of experimentation

(A) Body weight				
	NS	HS	TL	TH
Body weight (g)				
Initial	275.0 ± 4.2	271.9 ± 3.8	264.0 ± 3.2	269.9 ± 2.8
Final	397.0 ± 5.9	366.9 ± 11.6	364.9 ± 10.4	395.1 ± 5.3

Abbreviations: NS, normal salt; HS, high salt; TL, HS + tadalafil (1 mg kg^−1^ day^−1^); TH, HS + tadalafil (10 mg kg^−1^ day^−1^).

**Table 3:** Heart rate and blood pressure (BP) at baseline and after 8 weeks of experimentation

	NS	HS	TL	TH
Heart rate (bpm)				
Initial	413.5 ± 9.2	429.6 ± 5.2	419.5 ± 7.9	421.0 ± 13.8
Final	374.2 ± 4.0	415.9 ± 15.2^*^	402.9 ± 15.6	383.8 ± 6.9
Systolic BP (mmHg)				
Initial	114.7 ± 1.8	116.0 ± 4.1	123.3 ± 4.6	112.4 ± 3.7
Final	120.9 ± 1.6	216.5 ± 7.1^**^	200.0 ± 8.9^**^	179.4 ± 3.1^**,##,†^
Mean BP (mmHg)				
Initial	94.8 ± 3.4	96.2 ± 3.9	100.1 ± 4.7	91.4 ± 3.6
Final	103.3 ± 1.9	189.8 ± 7.0^**^	173.5 ± 4.5^**^	156.4 ± 1.8^**,##^
Diastolic BP (mmHg)				
Initial	85.0 ± 4.2	86.3 ± 4.0	88.3 ± 5.1	81.1 ± 3.7
Final	94.3 ± 2.4	176.3 ± 7.2^**^	160.3 ± 4.2^**^	145.4 ± 1.5^**,##^

Abbreviations: NS, normal salt; HS, high salt; TL, HS + tadalafil (1 mg kg^−1^ day^−1^); TH, HS + tadalafil (10 mg kg^−1^ day^−1^).

* *p* < .05 and

** *p* < .01 vs. NS,

^#^ *p* < .05 and

^##^ *p* < .01 vs. HS,

^†^ *p* < .05 vs. TL.

### 3.2 Kidney function

**Figure 1:**
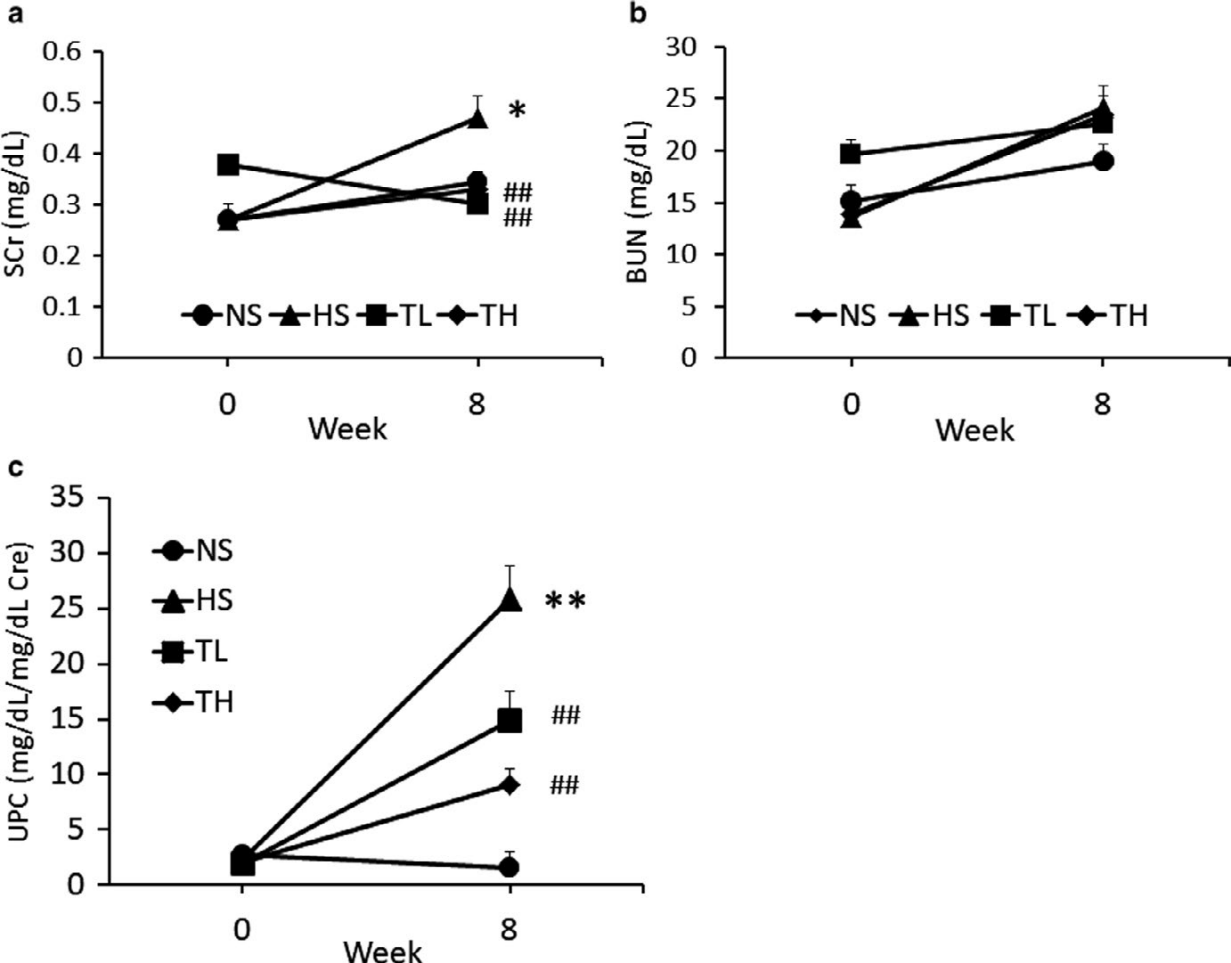
Changes in kidney function 8 weeks following treatment. (a) SCr, (b) BUN, and (c) urinary protein to creatinine ratio. NS, normal salt; HS, high salt; TL, HS + tadalafil (1 mg kg^−1^ day^−1^); TH, HS + tadalafil (10 mg kg^−1^ day^−1^). *p < .05 and **p < .01 vs. NS, ^#^ *p* < .05 and ^##^ *p* < .01 vs. HS

Changes in kidney function parameters are shown in Figure 1. After 8 weeks, the SCr was significantly higher in the HS group than that in the NS group (NS, 0.34 ± 0.02 vs. HS, 0.47 ± 0.04 mg/dl, Figure 1a), while the SCr in the TL and TH groups was not significantly different from that in the NS group (TL, 0.30 ± 0.03; TH, 0.33 ± 0.02 mg/dl). No significant differences in BUN levels were observed (Figure 1b). The HS group displayed significantly higher UPC levels than that in the NS group at 8 weeks (NS, 1.61 ± 0.17 vs. HS; 26.78 ± 3.07, Figure 1c), while the TL and TH groups had significantly lower UPC levels than those of the HS group at 8 weeks (TL, 14.87 ± 2.58; TH, 9.11 ± 1.43). Kidney function parameters did not significantly differ between the NS group and the group that was fed a normal diet and treated with high‐dose tadalafil (Table S1b).

### 3.3 Glomerulosclerosis

**Figure 2:**
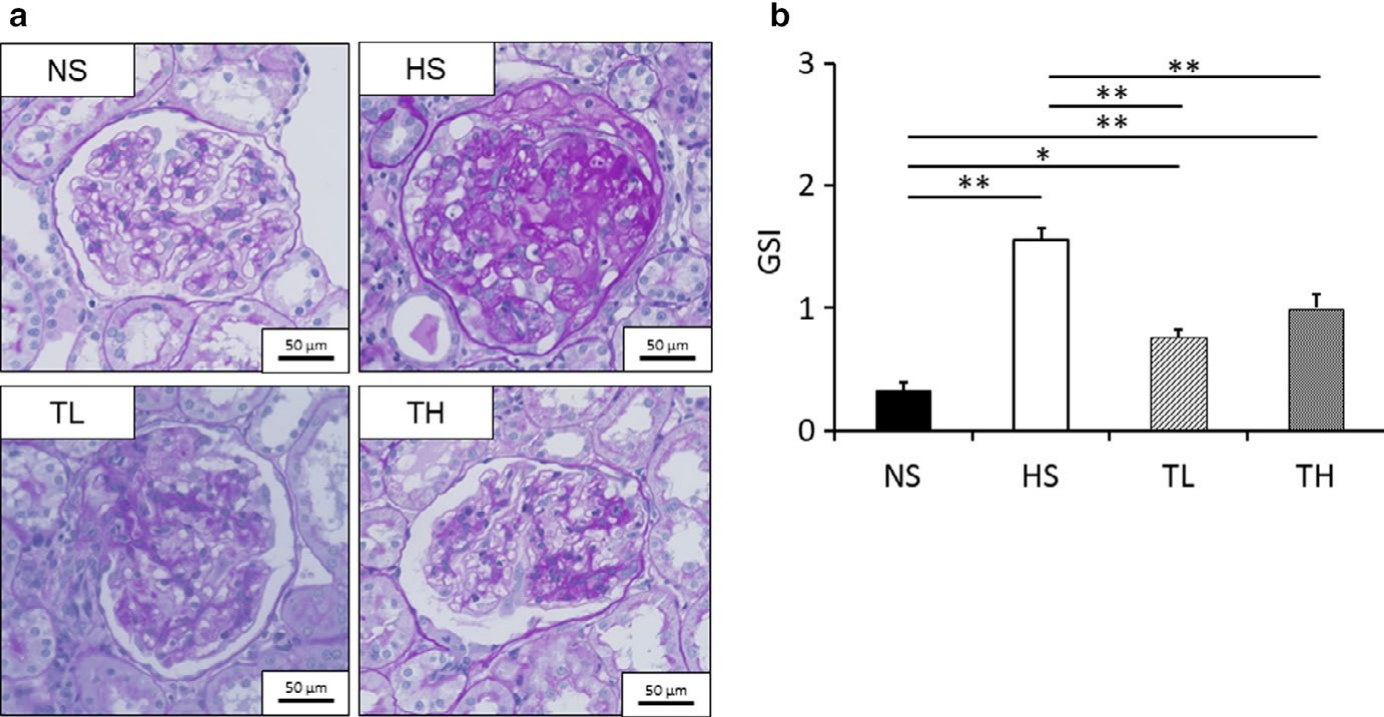
GSI determination. (a) PAS‐stained glomeruli from each group and (b) evaluation of the GSI. NS, normal salt; HS, high salt; TL, HS + tadalafil (1 mg kg^−1^ day^−1^); TH, HS + tadalafil (10 mg kg^−1^ day^−1^). *p < .05 and **p < .01 vs. NS

Representative PAS‐stained glomeruli from each group are shown in Figure 2a. The HS, TL, and TH groups had significantly greater GSIs than the NS group (*p* < .01, Figure 2b). However, the GSI was significantly less in the TL and TH groups than it was in the HS group.

### 3.4 Glomerular endothelial cells and podocyte foot processes

**Figure 3:**
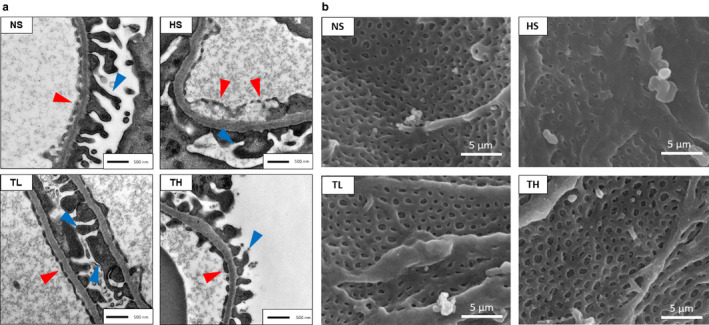
Glomeruli microstructure in each group. (a) Structure of the glomerular filtration barrier (red arrowheads, endothelial cells; blue arrowheads, podocyte foot processes). (b) Glomerular endothelial fenestrations. NS, normal salt; HS, high salt; TL, HS + tadalafil (1 mg kg^−1^ day^−1^); TH, HS + tadalafil (10 mg kg^−1^ day^−1^)

Transmission electron microscopy was used to observe the structure of the glomeruli, which consisted of endothelial cells, a glomerular basement membrane, and podocyte foot processes (Figure 3a). In the NS group, endothelial cells were observed lining the glomerular basement membrane (Figure 3a, red arrowheads). However, in the HS group, we observed detachment of the endothelial cells from the glomerular basement membrane and foot process effacement (Figure 3a, red and blue arrowheads, respectively). Both the TL and TH groups displayed attenuation of these effects. Scanning electron microscopy revealed fenestrations on the glomerular endothelial cells (Figure 3b), which were less evident in the HS group. However, the TL and TH groups maintained these fenestrations, despite the high‐salt diet (Figure 3b).

### 3.5 Renal fibrosis

**Figure 4:**
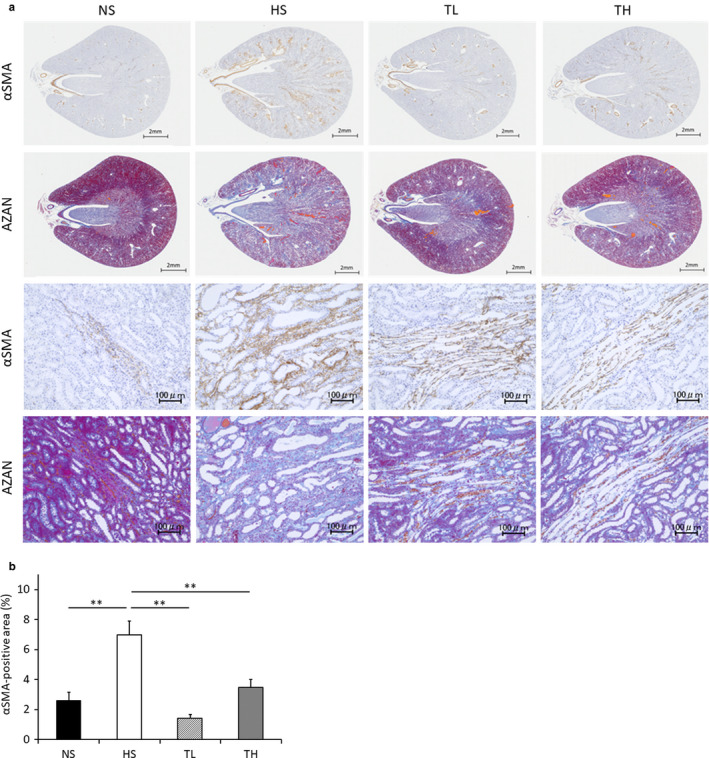
Renal fibrosis in each group. (a) The top two rows depict the entire kidney; the bottom two rows show zoomed images. (b) Evaluation of αSMA‐positive areas. NS, normal salt; HS, high salt; TL, HS + tadalafil (1 mg kg^−1^ day^−1^); TH, HS + tadalafil (10 mg kg^−1^ day^−1^). *p < .01 vs. NS

Figure 4a shows αSMA‐immunostained and Azan‐stained kidney tissue sections from each group. αSMA expression corresponded to areas with increased collagen accumulation, as indicated by Azan staining. The αSMA‐positive area was significantly larger in the HS group than in the NS group and significantly smaller in the TL and TH groups than in the HS group (Figure 4b).

### 3.6 PAI1 mRNA expression

**Figure 5:**
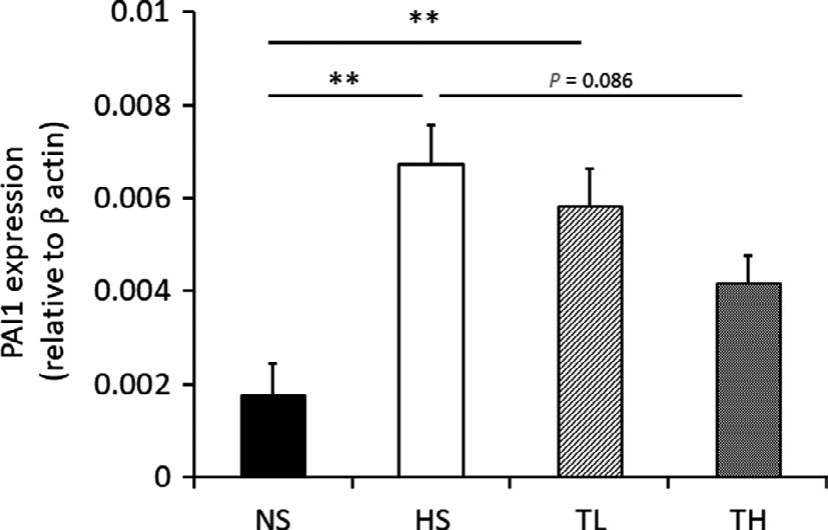
PAI1 mRNA expression in kidneys. PAI1 mRNA expression in kidney tissues after 8 weeks of treatment. NS, normal salt; HS, high salt; TL, HS + tadalafil (1 mg kg^−1^ day^−1^); TH, HS + tadalafil (10 mg kg^−1^ day^−1^). **p < .01 vs. NS

PAI1 mRNA expression was significantly greater in the HS group than in the NS group (Figure 5). The TL and TH groups displayed dose‐dependent improvements in PAI1. However, these improvements were not significantly different from the PAI1 level in the HS group (Figure 5).

## 4 DISCUSSION

Our results demonstrate that tadalafil, a PDE5 inhibitor, had renoprotective effects in a CKD rat model. Antihypertensive agents, such as angiotensin‐converting‐enzyme inhibitors, angiotensin Ⅱ receptor blockers, and calcium channel blockers, are commonly used for renal dysfunction related to hypertension and are considered renoprotective for the amelioration of glomerular hyperfiltration. However, some of these agents are ineffective in improving proteinuria even with a reduction in BP (Takai et al., 2010; Yao et al., 2003). In this study, tadalafil attenuated proteinuria, SCr levels, and kidney tissue injuries such as fibrosis and glomerulosclerosis, similar to other antihypertensive agents; even low‐dose tadalafil displayed significant renoprotective effects while exhibiting no significant BP‐lowering activity.

Moreover, the renoprotective effects of tadalafil may stem from enhanced protection of the glomerular structures against fibrosis. Tadalafil attenuated proteinuria caused by glomerular injury, diminished glomerulosclerosis, and maintained glomerular structure. The mechanisms underlying these effects of tadalafil remain to be elucidated, but PDE5 expression in the podocyte foot process has been previously reported (Dousa, 1999; Sonneveld et al., 2017). Podocytes, the basement membrane, and the glomerular endothelium play an important role, forming the glomerular filtration barrier. Crosstalk between the endothelium and podocytes is important for glomerular function (Hodgin et al., 2013; Sun et al., 2013). Considering these insights, PDE5 and related cGMP signaling might be involved in both endothelial and podocyte function. Therefore, PDE5 inhibition likely preserved glomerular filtration function. Further studies are warranted to confirm this hypothesis and determine whether tadalafil directly affects podocytes.

Fibrosis is a commonly observed pathological change during kidney dysfunction. Recently, PAI1 was shown to induce fibroblast activation and αSMA expression (Pincha et al., 2018). In this study, PAI1 mRNA expression was upregulated in the kidneys of hypertensive rats, which was dose‐dependently improved in tadalafil‐treated rats. These results indicate that tadalafil might prevent the increased myofibroblasts observed during fibrosis by inhibiting PAI1 upregulation.

Our preliminary study revealed that a normal‐diet group treated with tadalafil did not show any differences in kidney function parameters relative to those in the NS group. This suggested that tadalafil suppressed or prevented renal dysfunction but did not enhance renal function. PDE5 is primarily expressed in smooth muscle cells, and thus, might be involved in renal hemodynamic regulation. The systolic BP was not affected by low‐dose tadalafil treatment. However, its effects on renal blood flow and glomerular hyperfiltration remain unclear. In this study, we used the tail‐cuff method to measure BP, which has low sensitivity. Thus, we cannot definitively conclude that the renoprotective effects of tadalafil were independent of its BP‐lowering effects. In fact, αSMA expression in the outer medulla, which is the most easily damaged by high BP‐induced stress, was obviously attenuated by tadalafil treatment. Further investigations of the mechanisms underlying the renoprotective effects of PDE5 inhibitors could create new therapeutic targets.

There were some limitations to this study. First, different trends were observed in serum electrolytes between the TL and TH groups. The reason for this remains unclear, but it may be owing to variations among individuals at baseline. Ideally, the cutoff value used would have been set at the onset of the experiment. However, this does not challenge the observed renoprotective effects of tadalafil. Second, PDE5 inhibitors can have adverse effects, which should be considered. Clinically, tadalafil can cause a sudden and pathological decrease in BP (Kloner et al., 2003). It can also decrease blood flow to the optic nerve, causing sudden vision loss (Peter, Singh, & Fox, 2005). This effect could more likely occur in a subset of patients with heart disease, diabetes, and hypertension, who are prescribed tadalafil. Finally, there are potential interactions between high‐salt diet and tadalafil administration. This should be investigated statistically using two‐way analysis of variance. Furthermore, we had fed Dahl salt‐sensitive rats a high‐salt diet over 8 weeks, which is a longer period than what has been previously reported (Feng et al., 2017), since, in our preliminary study, 4 weeks of exposure did not increase SCr or induce kidney injury.

## 5 CONCLUSIONS

We have demonstrated that tadalafil has promising effects on CKD with high‐salt‐induced hypertension and that mechanistically, these effects may be associated with the regulation of PAI1 and myofibroblast levels and the protection of glomerular structures. This suggests that PDE5 inhibitors may help to prevent the progression of CKD with hypertension and other complications into ESKD.

## AUTHOR CONTRIBUTIONS

N.T., Y.H., K.K. conceived and designed the study; N.T., Y.H., A.N., K.H., Y.M., S.T., contributed for the acquisition of data. N.T., Y.H., T.K., analyzed the data; N.T., Y.H., A.N., S.T., K.K. drafted the article; N.T., Y.H. revised the manuscript for intellectual content; N.T., Y. H. approved the final version of the paper. All authors approved the manuscript and agreed to be accountable of the manuscript.

## Supporting information



Table S1
