# Supplementary material for: The phosphodiesterase 5 inhibitor tadalafil has renoprotective effects in a rat model of chronic kidney disease
Source: Physiol Rep. 2020 Sep 5;8(17):e14556. doi: 10.14814/phy2.14556 (PMC7503090; doi:10.14814/phy2.14556)
Supplement: Supplementary file 1 — Table S1 [file PHY2-8-e14556-s001.docx]

Supplementary Table 1A: SCr, BUN and UPC in normal plus tadalafil 10 mg/kg group

|  |  | NT |
| --- | --- | --- |
| SCr (mg/dL) | Initial | 0.3 ± 0.0 |
|  | Final | 0.3 ± 0.0 |
| BUN  (mg/dL) | Initial | 19.2 ± 0.5 |
|  | Final | 19.3 ± 0.4 |
| UPC | Initial | 1.3 ± 0.2 |
|  | Final | 1.4 ± 0.2 |

1B: Heart rate, SBP, MBP, and DBP in normal plus tadalafil 10 mg/kg group

|  |  | NT |
| --- | --- | --- |
| Heart rate  (bpm) | Initial | 395.8 ± 4.6 |
|  | Final | 366.2 ± 9.5 |
| Systolic BP  (mmHg) | Initial | 120.6 ± 2.2 |
|  | Final | 127.9 ± 2.8 |
| Mean BP  (mmHg) | Initial | 98.3 ± 2.9 |
|  | Final | 107.2 ± 3.5 |
| Diastolic BP  (mmHg) | Initial | 87.2 ± 3.6 |
|  | Final | 97.0 ± 4.0 |

NT; normal salt + tadalafil (10 mg·kg^−1^·day^−1^)
